# Research on Multi-Level Scheduling of Mine Water Reuse Based on Improved Whale Optimization Algorithm

**DOI:** 10.3390/s22145164

**Published:** 2022-07-10

**Authors:** Lei Bo, Zhihan Li, Yang Liu, Yuangan Yue, Zihang Zhang, Yiying Wang

**Affiliations:** 1 School of Mechanical Electronic & Information Engineering, China University of Mining and Technology (Beijing), Beijing 100083, China; bolei@student.cumtb.edu.cn (L.B.); zhihan_li@student.cumtb.edu.cn (Z.L.); zqt2000403075@student.cumtb.edu.cn (Y.Y.); zhangzh@student.cumtb.edu.cn (Z.Z.); 2 School of Mechanical and Equipment Engineering, Hebei University of Engineering, Handan 056038, China; wangyiying@hebeu.edu.cn

**Keywords:** mine water reuse, reuse efficiency, whale optimization algorithm, opposition-based learning, Levy flight, nonlinear convergence factor, adaptive inertia weight, efficient utilization of deployment

## Abstract

Aiming at the problem of the inefficiency of coal mine water reuse, a multi-level scheduling method for mine water reuse based on an improved whale optimization algorithm is proposed. Firstly, the optimization objects of mine water reuse time and reuse cost are used to establish the optimal scheduling model of mine water. Secondly, in order to overcome the defect that the whale optimization algorithm (WOA) is prone to local convergence, the opposition-based learning strategy is introduced to speed up the convergence speed, the Levy flight strategy is used to enhance the ability of the algorithm to jump out of the local optimization, the nonlinear convergence factor is used to balance the global and local search ability, and the adaptive inertia weight is used to improve the optimization accuracy of the algorithm. Finally, the improved whale optimization algorithm (IWOA) is applied to the mine water optimization scheduling model with multiple objects and constraints. The results show that the reuse efficiency of the multi-level scheduling method of mine water reuse is increased by 30.2% and 31.9%, respectively, in the heating and nonheating seasons, which can significantly improve the reuse efficiency of mine water and realize the efficient utilization of mine water reuse deployment. At the same time, experiments show that the improved whale optimization algorithm has higher convergence accuracy and speed, which proves the feasibility and superiority of its improvement strategies.

## 1. Introduction

As a by-product of coal mining, mine water is mainly derived from groundwater, water used for equipment during production, and surface infiltration water [1,2]. Relevant statistics show that the annual production of mine water in China is about 7 billion m^3^, which is a huge output. In addition to depending on the original composition of groundwater, mine water is also contaminated by dust in mines and various emissions from the mining process [3]. Mine water can be turned into various water resources needed for many scenarios after treatment, such as water for production, greening, and domestic use. Therefore, it has great potential for utilization [4]. At present, the reuse efficiency of mine water resources in China is low, the comprehensive utilization capacity is insufficient, and the integration into the unified allocation of water resources is not enough [5]. How to realize the utilization of mine water resources has become an urgent problem to be solved.

At present, research has been conducted by scholars on how to improve the level of mine water resource utilization and achieve its efficient management and deployment, and they have achieved good results. Reference [6] proposed a finite element groundwater management optimization framework to solve the conflict between mine drainage, water supply, and environmental protection. Reference [7] proposed the relevant theories and technologies of coal mine underground reservoirs and realized optimal scheduling according to the relationship between water supply and demand, which improved the utilization rate of mine water. Reference [8] proposed a two-layer optimization model under uncertain conditions based on an equilibrium strategy to solve the “coal-water” contradiction and promote the sustainable development of coal fields. Reference [9] proposed a comprehensive evaluation system based on the PSO-AHP model, which provided a reference for reflecting the sustainable development capacity of mine water resources and reducing the adverse impact of sewage discharge on the environment. Reference [10] proposed an improved genetic algorithm–particle swarm optimization algorithm to achieve the optimal allocation of water resources in the Pingshuo mining area. Reference [11] realized energy-saving scheduling of mine drainage system based on exponential moving average method and particle swarm algorithm. Reference [12] developed an automatic monitoring system for mine water purification and treatment, which ensures the quality of mine water, reduces treatment costs, and improves operational efficiency. In addition to the above-mentioned research on the management and deployment of mine water treatment and discharge, research on mine water reuse and scheduling has gradually emerged in recent years, which is a new problem of coordination between the coal mine environment and economic development under the combined effect of mine water resource utilization and rapid development of communication technology. Reference [13] used the improved particle swarm algorithm to solve the optimal scheduling model of the mine water reuse system and verified that the scheduling scheme obtained can improve the mine water reuse rate and system operation efficiency. Reference [14] proposed a mine water reuse information processing framework based on edge computing and used the particle swarm algorithm to achieve optimal scheduling, which effectively improved the operating efficiency of the processing system. Reference [15] designed a mathematical model for the reuse of mine water in order to achieve the optimal configuration of mine water. It realized the adaptive improvement of the GA-PSO hybrid optimization algorithm, so as to improve the performance of the mine water reuse system, and confirmed the superiority of the improved algorithm. At present, the optimal scheduling problem of mine water is still in the exploratory stage. There is no mature research that completely solves the optimization problems such as efficiency and cost in mine water scheduling.

At present, the mine water of most coal mines adopts the method of nearby reuse or unified reuse. These traditional reuse methods are simple and convenient to operate, but will cause problems such as a slow mine water reuse rate. The “14th Five-Year Plan for the Construction of a Water-Saving Society” emphasizes the implementation of unconventional water source utilization projects such as mine water, accelerates the comprehensive utilization of coal mine water on a large scale, and realizes leveled treatment and quality utilization. It confirms the importance of optimizing the configuration of mine water treatment and reuse and improving production efficiency. Usually, the improvement of production efficiency lies in the improvement of efficiency and the optimization of cost. It often presents the characteristics of nonlinear, multiple objectives, and multiple constraints in the process of realizing optimal scheduling. For such problems, the swarm intelligence algorithm is usually used to solve them; for an example, Reference [16] used intelligent algorithms to model and analyze the fault prediction of water distribution networks. For this reason, this paper introduces the whale optimization algorithm, which was proposed by Mirjalili et al. in Australia in 2016 [17]. Compared with other swarm intelligence algorithms, it has the characteristics of less parameters, simple calculation, and fast convergence speed [18] and has been initially applied in coal mine inrush water resource prediction [19,20], water resource scheduling [21], and so on. Furthermore, for the scenario of mine water reuse and scheduling, the whale optimization algorithm also has good adaptability.

The main research contents of this paper are as follows:

(1) The mine water reuse system is introduced, and a multi-level scheduling method for mine water reuse is proposed based on the characteristics of the mine water reuse treatment process.
(2) The optimal scheduling model of mine water is established with mine water reuse efficiency and cost as optimization goals.
(3) Aiming at the defect that the basic whale optimization algorithm is prone to local convergence, opposition-based learning, Levy flight, nonlinear convergence factor, and adaptive inertia weight are introduced to realize the hybrid strategy improvement.
(4) Combining the improved algorithm with the optimal scheduling model, and through simulation experiments, the efficiency of the multi-level scheduling method and the superiority of the improved whale optimization algorithm are confirmed.

The structure of this paper is as follows. Section 2 describes the mine water reuse system and proposes a multi-level reuse scheduling strategy. Section 3 describes the establishment of the mathematical model. Section 4 introduces the whale optimization algorithm and its hybrid strategy improvement scheme. Section 5 describes the realization of mine water scheduling method based on an improved algorithm. Section 6 is the conclusion of the work of this paper.

## 2. Mine Water Reuse System

### 2.1. Mine Water Reuse Treatment Process

**Figure 1:**
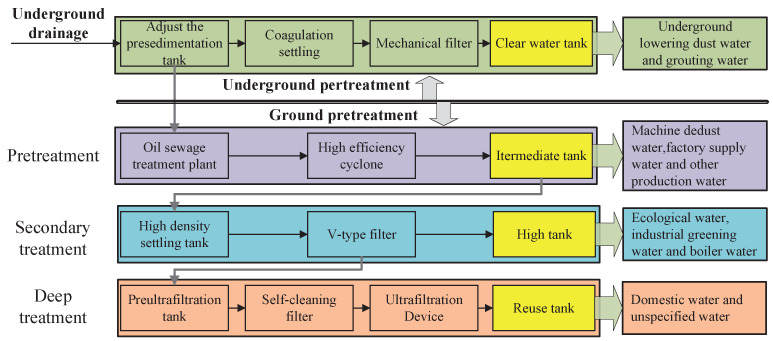
Mine water process flow with graded and fractionated utilization.

Taking a coal mine in the Inner Mongolia Autonomous Region of China as an example, its mine water treatment process is shown in Figure 1. The mine water treatment process consists of three levels of treatment, namely pretreatment, secondary treatment, and deep treatment. The quality of mine water is continuously improved with the increase of treatment levels. After the mine water flows into the pre-sedimentation tank, part of it is pretreated by the coagulation sedimentation device and mechanical filter and then flows into the clear water tank. The reused water in the clear water tank can meet the requirements of underground water with lower dustand grouting water, so as to realize on-site reuse. The mine water after ground pretreatment, secondary treatment, and advanced treatment is stored in the intermediate tank, high tank, and reuse tank, respectively [22]. Among them, the intermediate tank can be used for machine dust water, factory water, and other production water supply, the high tank is responsible for providing ecological water, industrial greening water, and boiler water, and the reuse tank provides high-quality mine water, which can be used for ecological, agricultural, living, and other purposes with high water quality requirements [23]. Based on a comprehensive analysis, this mine water reuse treatment process can realize the classification treatment and quality utilization of mine water.

### 2.2. Multi-Level Scheduling of Mine Water Reuse

**Figure 2:**
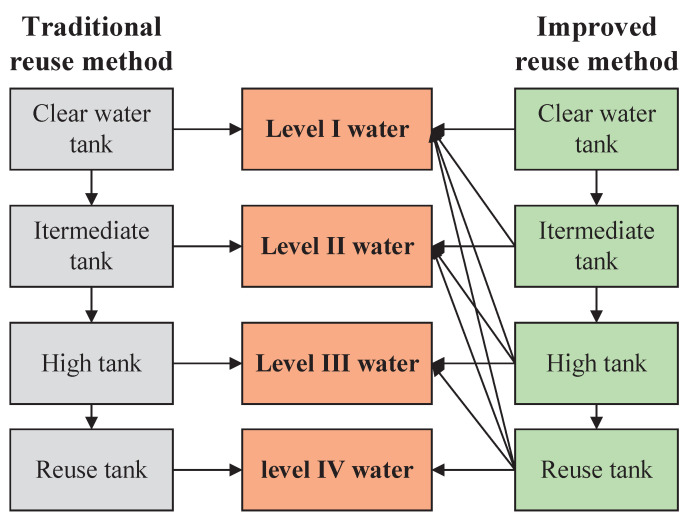
Schematic diagram of multi-level scheduling of mine water reuse.

According to the water quality characteristics of the four reuse water points in the mine water reuse treatment process, the mine water points are correspondingly divided into four levels. In order to solve the problems of the slow recycling rate and low production efficiency caused by traditional recycling methods, this paper proposes a multi-level scheduling method for mine water recycling, as shown in Figure 2. The tank with high treatment levels can supply water to their corresponding water points of the same level or lower levels. Therefore, for low-level water points, there are multiple tanks supplying water to them at the same time. The novelty of this method is that it breaks the barriers between different levels of treatment links and water points in the traditional mine water reuse method and interconnects them, thereby enhancing the integrity of the mine water reuse system and greatly improving the efficiency of mine water reuse. Since there is a linear treatment relationship among the four water tanks, which satisfies the water balance constraints, the water volume change of a single water point will inevitably affect the water distribution relationship of the entire treatment system. This feature provides the feasibility of the physical logic of mine water scheduling optimization.

## 3. Mine Water Scheduling Model

### 3.1. Objective Function

According to the optimization objectives of mine water reuse time and reuse cost, the corresponding objective function is established as follows:

(1) $$\min f\left(C_{1},C_{2},\;...,\;C_{M}\right)=\omega_{1}\frac{\sum_{i=1}^{M}Q_{i}C_{i}}{Q_{\max}}+\omega_{2}\frac{\max(\frac{C_{1}}{v_{1}},\frac{C_{2}}{v_{2}},\;...,\;\frac{C_{M}}{v_{M}})}{T_{\max}}$$

*M* represents the number of mine reuse water points:

(1) $Q_{i}$ represents the treatment cost per ton of water, and $C_{i}$ represents the amount of mine water reused at the *i*-th reuse water point. Therefore, $Q_{i}C_{i}$ represents the total treatment cost of the *i*-th reuse water point. The treatment cost of each reuse water point is used as the mine water reuse cost.

(2) $v_{i}$ represents the mine water treatment speed of the *i*-th reuse water point, so $C_{i}/v_{i}$ represents the mine water treatment time of the *i*-th reuse water point. In order to meet the demand of water points, it is necessary to ensure that each water reuse point completes the processing and scheduling, so the maximum processing time is taken as the mine water reuse time.

(3) $Q_{\max}$ and $T_{\max}$ represent the maximum cost and maximum time in the process of mine water reuse, and their expressions are as follows:

(2) $$Q_{\max}=\max\left(Q_{1},Q_{2},\;...,\;Q_{M}\right)\cdot \sum_{i=1}^{M}C_{i}$$

(3) $$T_{\max}=\frac{\sum_{i=1}^{M}C_{i}}{v_{\min}}$$

When all the reused water is taken from the tank with the highest cost, the cost at this time is determined to be the maximum cost of the mine water reuse process. When all the reused water is taken from the tank with the lowest treatment speed, the time is determined as the maximum time of the mine water reuse process. The reuse cost rate and reuse time rate are obtained by dividing the reuse cost and reuse time by their maximum value, thereby normalizing the two optimization objectives to the same order of magnitude. Such a mathematical process can normalize the two optimization objectives to the same order of magnitude.

(4) $\omega_{1}$ and $\omega_{2}$ represent the weight coefficient of the reuse time and reuse cost, respectively. Usually, the reuse cost and reuse time have a restrictive relationship. Therefore, in the optimal scheduling of mine water, both will not be optimal at the same time. The value of $\omega_{1}$ is often greater than the value of $\omega_{2}$. In this paper, in order to improve the efficiency of mine water reuse, the weights of the two indicators are equal, that is $\omega_{1}=\omega_{2}=0.5$.

### 3.2. Restrictive Condition

(1) Balance of water supply and demand:

(4) $$\sum_{i=1}^{M}C_{i}=S\cdot D=R$$

In the formula, $C_{i}$ represents the water consumption of the *i*-th water point, *S* represents the underground water inflow volume of the mine, *D* represents the mine water reuse rate, and *R* represents the demand of the mine water point.

(2) Water supply capacity restriction:

(5) $$C_{i\;\min}\leq C_{i}\leq C_{i\;\max}$$

In the formula, $C_{i}$ represents the water supply volume of the *i*-th treatment link of mine water, $C_{i\;\min}$ represents the minimum water supply volume of the *i*-th processing link, and $C_{i\;\max}$ represents the maximum water supply volume of the *i*-th processing link.

(3) Processing speed restriction:

(6) $$V_{i\;\min}\leq V_{i}\leq V_{i\;\max}$$

In the formula, $V_{i}$ represents the processing speed of the *i*-th processing link of mine water. $V_{i\;\min}$ represents the minimum processing speed of the *i*-th processing stage, and $V_{i\;\max}$ represents the maximum processing speed of the *i*-th processing stage.

(4) Processing cost restriction:

(7) $$Q_{i\;\min}\leq Q_{i}\leq Q_{i\;\max}$$

In the formula, $Q_{i}$ represents the treatment cost of the *i*-th treatment link of mine water, $Q_{i\;\min}$ represents the minimum processing cost of the *i*-th processing stage, and $Q_{i\;\max}$ represents the maximum processing cost of the *i*-th processing stage.

## 4. Implementation of the Improved Whale Optimization Algorithm

### 4.1. Basic Whale Optimization Algorithm

Reference [24] proposed the calculation principle and mathematical expression of the whale optimization algorithm, which mainly includes three stages: search prey, surrounding prey, and bubble net prey.

(1) Search prey: This refers to the process in which whales swim randomly and search for prey in space when they hunt. The mathematical expression for this process is as follows:

(8) $$\left\{\begin{array}{c} X\left(t+1\right)=X_{\mathrm{rand}\;}-A\cdot D\\ D=\left|C\cdot X_{\mathrm{rand}\;}-X\left(t\right)\right| \end{array}\right.$$

where *t* represents the number of current iterations, representing a random individual in the current whale population, *A* and *C* are constant vectors, and the relevant calculation expressions are as follows:

(9) $$A=2a\cdot r_{1}-a$$

(10) $$C=2\cdot r_{2}$$

(11) $$a=2-2t/t_{\max}$$

where *a* is the convergence factor, $r_{1}$ and $r_{2}$ are random vectors in [0,1], and $t_{\max}$ is the maximum number of iterations.

(2) Surrounding prey: This refers to the process that the whale population revolves around the target, that is the best individual. Its mathematical expression is as follows:

(12) $$\left\{\begin{array}{c} X\left(t+1\right)=X{}^{*}\left(t\right)-A\cdot D\\ D=\left|C\cdot X{}^{*}\left(t\right)-X\left(t\right)\right| \end{array}\right.$$

where $X^{*}\left(t\right)$ represents the location information of the best individual whale.

(3) Bubble net prey: This refers to the process in which the whale continuously exhales air bubbles to drive the prey to the center of the spiral in the stage of spiral encircling. Its mathematical expression is as follows:

(13) $$\left\{\begin{array}{c} X\left(t+1\right)=X{}^{*}\left(t\right)+D\cdot e^{bl}\cdot \cos\left(2\pi l\right)\\ D=\left|X{}^{*}\left(t\right)-X\left(t\right)\right| \end{array}\right.$$

where *D* represents the distance between the currently selected whale individual and the best individual in the population, *b* is the constant that determines the shape of the spiral curve, and *l* is a random number between [−1,1].

In the process of iterative calculation, the group of whales decides the optimization stage according to the random number p∈[0,1]. When p>0.5, the group of whales preys on the bubble net; when p<0.5, the group of whales further selects according to the value of $\left|A\right|$; when $\left|A\right|>1$, the group of whales searches and preys; when $\left|A\right|>1$, swarms of whales perform wrap-around predation.

### 4.2. Improved Whale Optimization Algorithm

The basic whale optimization algorithm is prone to fall into local optima in the iterative process, which affects the accuracy of the solution [25]. Therefore, in order to achieve a better optimization search effect, the following strategy improvements are implemented in this paper for the whale optimization algorithm:

(1) Opposition-based learning strategy: Opposition-based learning [26] makes trade-offs by comparing the adaptability of the current solution and its opposite solution, so as to solve the problem of slow convergence speed that may be caused by the randomness of the initial population assignment. According to the definition, in the [27], assuming that there is a point $\left(x_{1},x_{2},\;...,\;x_{n}\right)$ in the n-dimensional coordinate system, $x_{1},\;...,\;x_{n}$ are all real numbers and $x_{i}\in \left[a_{i},b_{i}\right]$, then define its inverse number: $\left({\tilde{x}}_{1},{\tilde{x}}_{2},\;...,\;{\tilde{x}}_{n}\right)$ as:

(14) $${\tilde{x}}_{i}=a_{i}+b_{i}-x_{i},i=1,\;...,\;n$$

Based on the opposition-based learning strategy, individuals are selected from the initial population location information $X=(x_{1},x_{2},\;...,\;x_{n})$, and their population opposite location information is established in turn according to the definition of the opposite solution to form the opposite population $\tilde{X}=\left({\tilde{x}}_{1},{\tilde{x}}_{2},\;...,\;{\tilde{x}}_{n}\right)$, which the fitness value of each individual is calculated separately. Sort and select the top n individuals as the initial population. This population initialization method using the opposition-based learning strategy ensures that the initial population is initialized randomly in the solution space and, at the same time, improves the adaptability to the objective function, which improves the convergence speed [28].

(2) Levy flight strategy: Levy flight is a method of simulating a creature’s random walk, a mechanism embodied in long-term short-distance walks, and occasional long-distance walks. The use of this walking mechanism in algorithm optimization can not only make the population perform a fine local search in the solution space, but also occasionally generate long-distance mutations, avoid falling into local optima, and improve the accuracy and ergodicity of the algorithm [29]. According to [30], the search formula based on the Levy flight strategy is as follows:

(15) $$X\left(t+1\right)=X\left(t\right)-\alpha \cdot s\cdot (X\left(t\right)-X_{rand})$$

(16) $$s=\frac{u}{{\left|v\right|}^{\frac{1}{\beta }}}$$

where α is the step size scaling factor, which is 0.01. *s* is the random step size of the Levy flight, where *u* and *v* satisfy the normal distribution, $u∼N(0,{\sigma_{u}}^{2})$, $v∼N(0,{\sigma_{v}}^{2})$, respectively. $\sigma_{u}$, $\sigma_{v}$ are defined as:

(17) $$\sigma_{u}={\left\{\frac{\Gamma \left(1+\beta \right)\cdot \sin\left(\frac{\pi \beta }{2}\right)}{\beta \cdot \Gamma \left(\frac{1+\beta }{2}\right)\cdot 2^{\frac{\beta -1}{2}}}\right\}}^{\frac{1}{\beta }}\;,\;\sigma_{v}=1$$

In the improvement of the whale optimization algorithm in this paper, the Levy flight strategy formula is used to replace the search prey Formula (8), which enhances the ability to jump out of the local optimum on the basis of achieving a better search effect.

**Figure 3:**
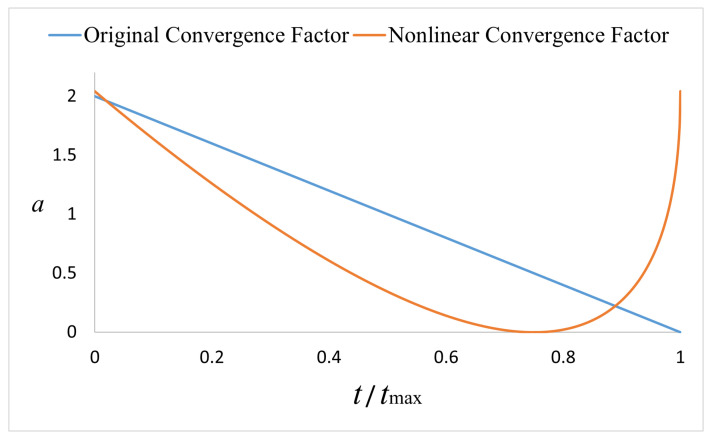
Nonlinear convergence factor.

(3) Nonlinear convergence factor: According to the optimization principle of the basic whale optimization algorithm, the search prey of the whale group represents the global search. Usually, the global search operation is only implemented in the early stage of the iterative calculation, which is determined by a linearly decreasing convergence factor [31]. When the convergence factor a≥1, global search or local search will be further selected according to the size of |A|. When a<1, only local search can be performed. In the original whale algorithm, *a* is completely greater than 1 in the early stage and completely less than 1 in the later stage, which results in the separation of the global and local search in the early stage and the later stage. Once the global optimal value is not found in the early stage, it is easy to fall into the local optimum in the later stage. In order to balance the global and local search capabilities in the whole iterative process, inspired by [32], a nonlinear convergence factor is introduced in this paper, and it is shown in Figure 3.

(18) $$a=8\cdot (e^{\sqrt{1-t/t_{\max}}-0.5}+e^{0.5-\sqrt{1-t/t_{\max}}}-2)$$

Compared with the linear convergence factor of the basic algorithm, the nonlinear convergence factor curve shows the characteristics of first slowly decreasing and then rapidly increasing. This improvement enables the algorithm in the early stage of the iteration to enter the local search mode earlier. In the later stage of the iteration, the value increases, which will make the algorithm perform a partial global search, thereby expanding the scope of optimization, so the improved convergence factor can balance the global and local search in the entire iterative process and has better iterative performance.

**Figure 4:**
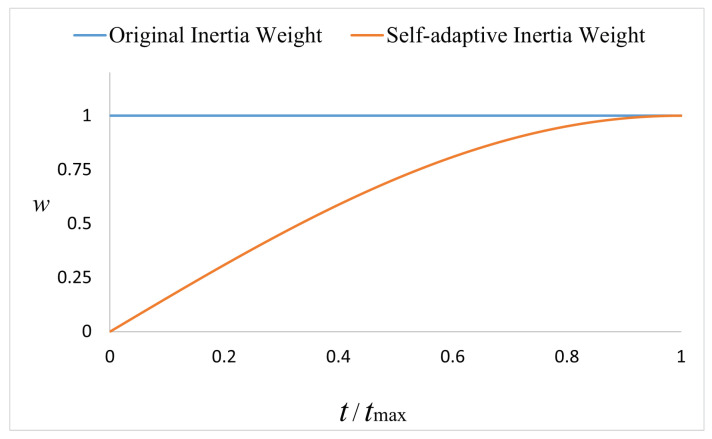
Self-adaptive inertia weight.

(4) Adaptive inertia weigh: The idea of inertia weight comes from the particle swarm algorithm, which represents the ability of the next generation of individuals to maintain the current state of individual motion and is an important parameter to adjust the algorithm’s search and optimization capabilities [33]. Inspired by this, the adaptive inertia weight is introduced in the improvement of the whale optimization algorithm in this paper, and it is shown in Figure 4.

(19) $$w\left(t\right)=\cos(\frac{\pi }{2}\cdot \left(1-\frac{t}{t_{\max}}\right))$$

At the beginning of the iteration, the weight *w* is small and the change speed is fast. Later, the value of the weight increases, but the change speed gradually slows down. Therefore, the adaptive inertia weight weakens the influence of the optimal individual on the position adjustment of the current individual in the early stage of the algorithm for global search, thus improving the global search capability. In the later stage, the weight gradually increases, so that the current individual can better inherit the optimal individual when the local search is performed, thus strengthening the local search capability [34]. Therefore, compared with the original inertia weight, which is always 1, the adaptive improvement method can dynamically adjust the iterative method according to the characteristics of different optimization stages, thereby improving the convergence accuracy. Combining the above strategies, the position update formula X(t+1) of the improved whale optimization algorithm is obtained as follows:

(20) $$\left\{\begin{array}{c} X\left(t\right)-\alpha \cdot s\cdot \left(X\left(t\right)-X_{rand}\right),p<0.5\cap \left|\vec{A}\right|>1\\ w\left(t\right)\cdot X{}^{*}\left(t\right)-A\cdot D,p<0.5\cap \left|\vec{A}\right|<1\\ w\left(t\right)\cdot X{}^{*}\left(t\right)+D\cdot e^{bl}\cdot \cos\left(2\pi l\right),p>0.5 \end{array}\right.$$

**Figure 5:**
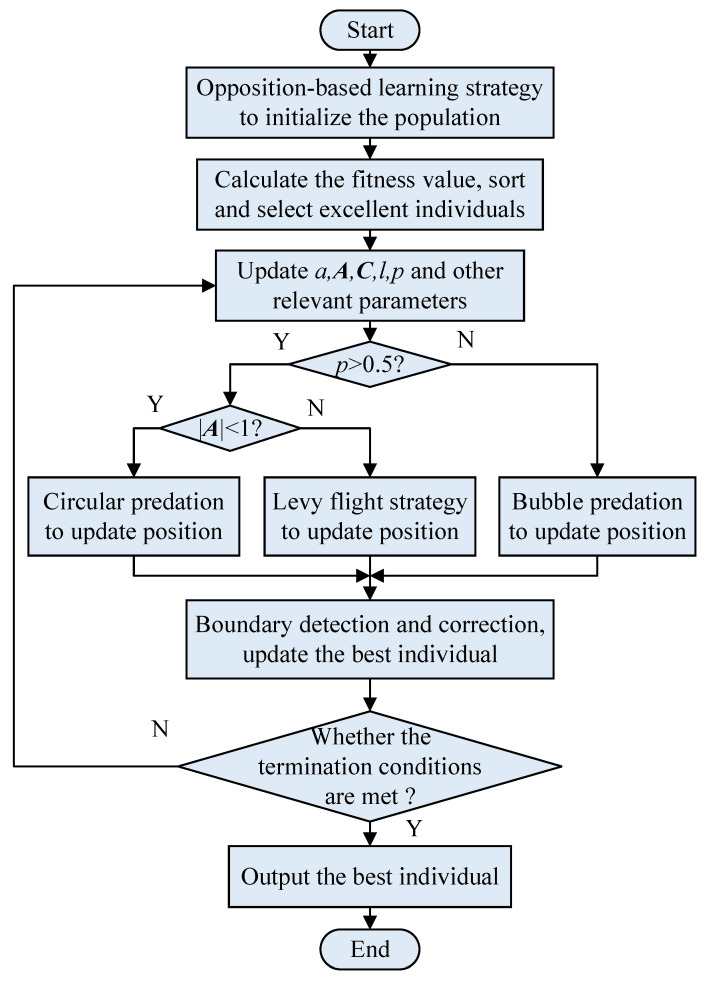
Flow chart of improved whale optimization algorithm.

According to the calculation process of the basic whale optimization algorithm, the flowchart of the improved whale optimization algorithm is shown in Figure 5.

### 4.3. Convergence Performance Test of Improved Whale Optimization Algorithm

In order to verify the performance of the improved whale optimization algorithm, test functions are introduced in this paper and compared with the basic whale optimization algorithm, genetic algorithm, and particle swarm algorithm for testing and analysis. The test functions used are shown in Table 1. Among them, the Sphere function is used to detect the convergence accuracy of the algorithm; the Ackley function is used to detect the convergence speed of the algorithm; the Rosenbrock function is used to detect the global search ability of the algorithm; the Alpine function is used to detect the local search ability of the algorithm; the Rastrigin function is used to detect the practicality of the algorithm in the case of a regular solution; the Griewank function is used to detect the ability of the algorithm to jump out of the local optimum.

**Figure 6:**
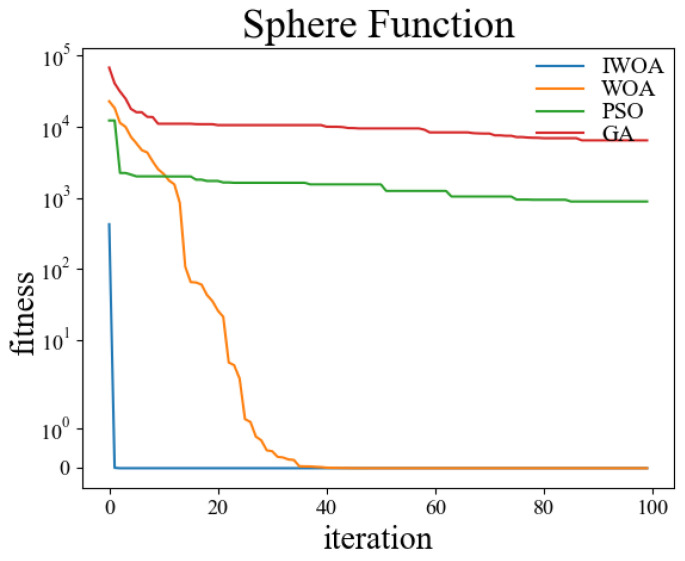
Sphere.

**Figure 7:**
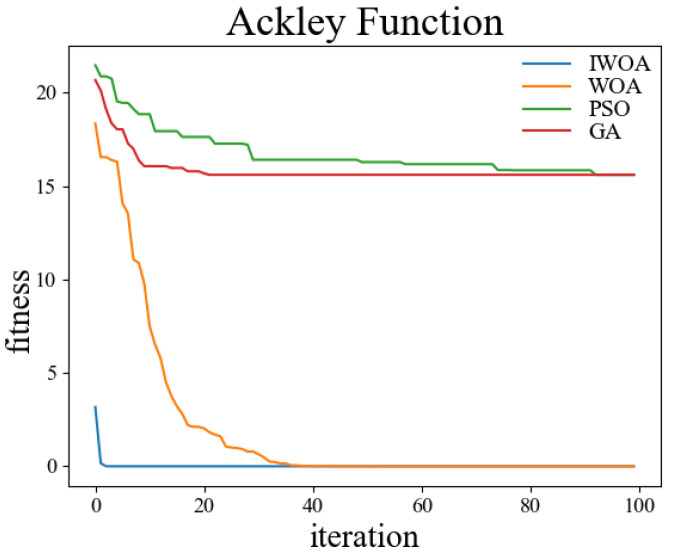
Ackley.

**Figure 8:**
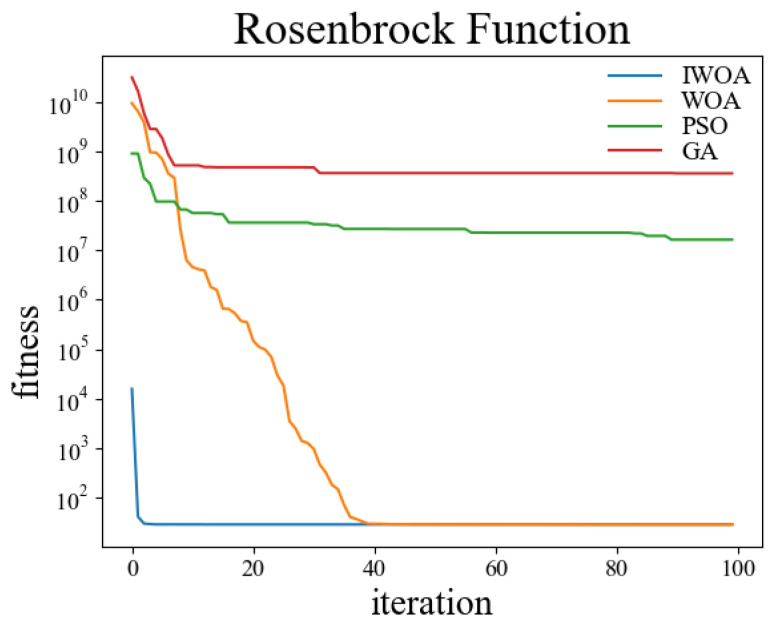
Rosenbrock.

**Figure 9:**
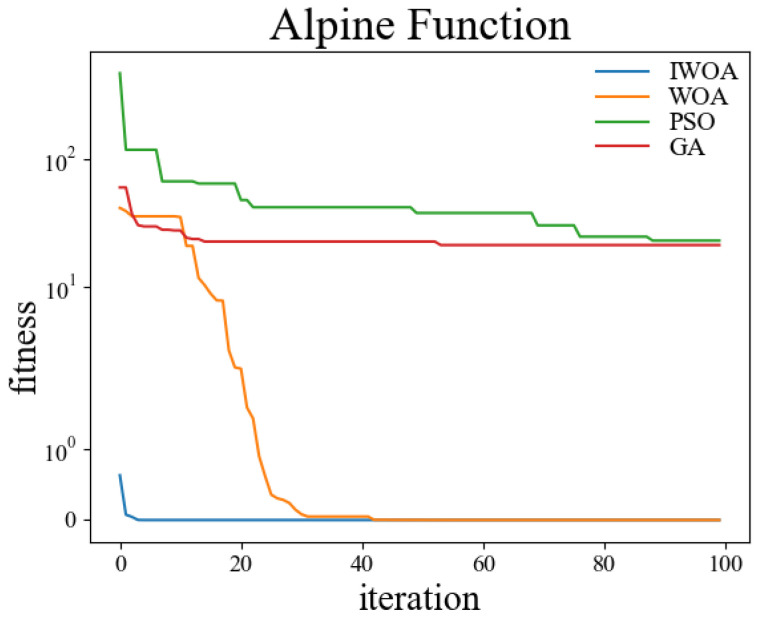
Alpine.

**Figure 10:**
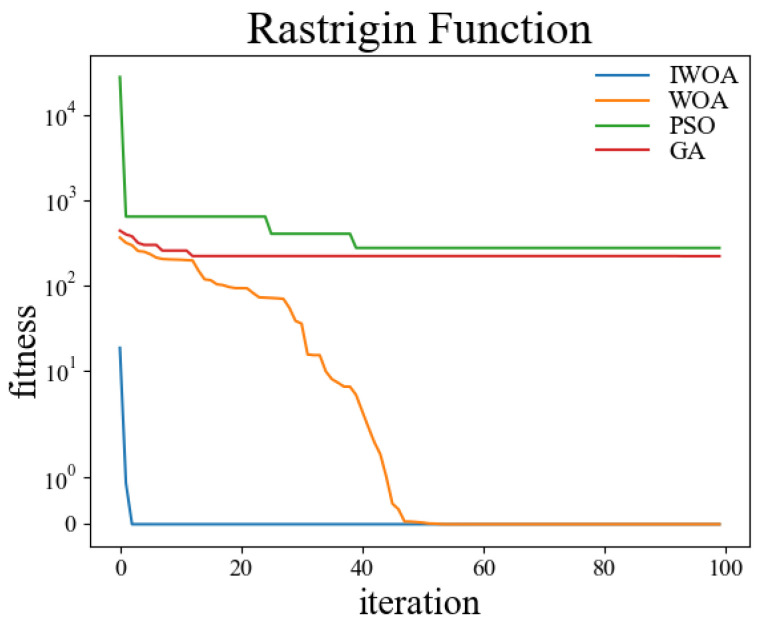
Rastrigin.

**Figure 11:**
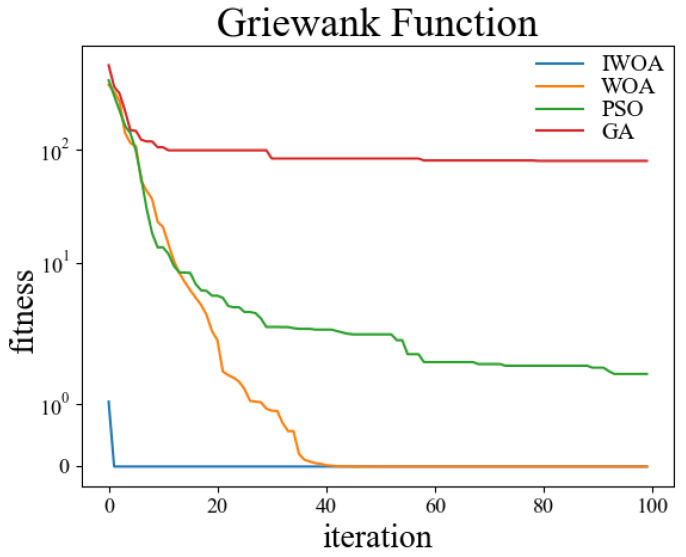
Griewank.

The test was carried out in a 30-dimensional search space. The population number was set to 50, and the maximum number of iterations was set to 100. Thirty independent experiments were carried out on the genetic algorithm, particle swarm optimization, whale optimization algorithm, and improved whale optimization algorithm, respectively. The crossover rate and variation rate of the GA were set to 0.8 and 0.1. The maximum particle velocity of PSO was set to 60. The spiral coefficient b of WOA and IWOA was set to 2. Table 2 shows the test results of the above four algorithms under the kinds of test functions, and the comparisons were made in terms of optimal solutions and standard deviations, respectively. The comparison figures of the change of the fitness values during the convergence of the four algorithms under each test function are shown in Figure 6, Figure 7, Figure 8, Figure 9, Figure 10 and Figure 11.

According to the test data and figures, it can be concluded that the WOA and IWOA have faster convergence speed and a better effect compared with the GA and PSO. Further analysis shows that, compared with the WOA, the IWOA greatly improved the optimal solution and standard deviation under the six test functions, thus overcoming the defect of local convergence to a greater extent.

Compared with the WOA, the optimal solution and standard deviation of the IWOA under the six test functions are greatly improved, and the defect of local convergence is overcome to a larger extent. After using the backward learning strategy, the initial population adaptation of the IWOA is greatly improved, and the number of convergences to the optimal solution is increased from several dozens to less than 10 times. After using the opposition-based learning strategy, the initial population adaptation is improved significantly, and the number of convergences to the optimal solution is increased from several dozens to less than 10. In summary, the IWOA has superior performance with higher convergence accuracy and speed. In summary, the IWOA has a superior performance with higher convergence accuracy and speed.

## 5. Optimization Realization of Mine Water Scheduling

### 5.1. Design Principle

**Figure 12:**
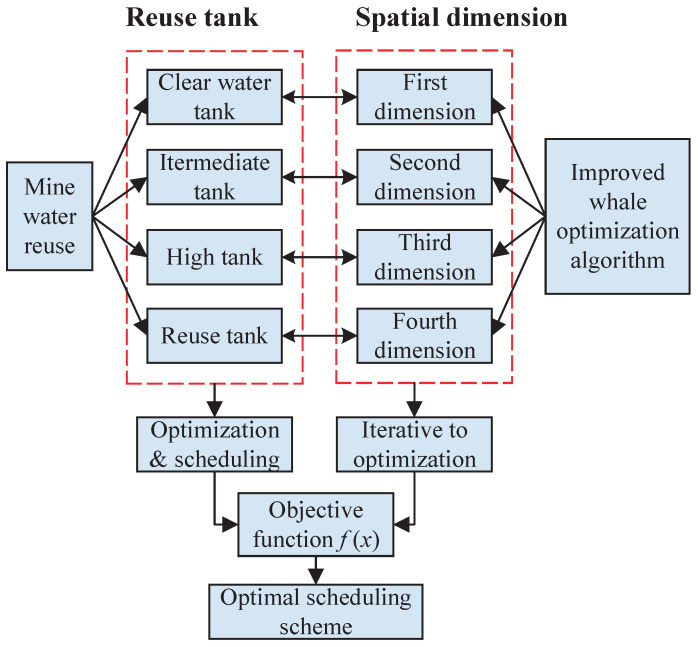
Schematic diagram of mine water scheduling based on improved whale optimization algorithm.

According to the spatial multi-dimensional characteristics of the improved whale optimization algorithm, this paper fully combines it with the multi-optimization objectives of mine water scheduling optimization. In this way, the transformation from complex problems to mathematical problems can be realized, that is the scheduling problem of mine water reuse tanks at all levels to water points at all levels is mapped to the optimization process of the whale optimization algorithm in the multi-dimensional solution space. The specific design principle is to combine each recycled water tank in the mine water reuse treatment process with the spatial dimension of the whale optimization algorithm. The physical meaning represented by each dimension value is the amount of water supplied by each tank to its water point. Iterative optimization is carried out under the constraints, and the optimal solution of the objective function based on mine water recycling efficiency and recycling cost is finally obtained. The combined schematic diagram is shown in Figure 12.

### 5.2. Mine Water Scheduling Operation Parameters

The object of mine water scheduling optimization is the water demand of multiple water points. In order to realize the efficient utilization and deployment of underground–surface water points, this paper comprehensively considers the characteristics of water consumption at all levels in the mining area and calculates the water consumption in the heating season and nonheating season, respectively, as shown in Table 3.

It can be observed that part of the mine drainage can be reused for dusting and grouting after underground treatment. Other mine water is transported to the ground and can be reused for production, ecological, and living water purposes such as machine dedust water, greening water, and boiler water after comprehensive treatment. In the whole mine water treatment and reuse, production water accounts for nearly 80%, showing the characteristics of low water quality requirements and concentrated water points. The water demand is basically stable except for the boiler water consumption in the heating season and the greening water consumption in the nonheating season. The treatment cost and treatment speed index of the mine water reuse treatment process are shown in Table 4.

### 5.3. Analysis of Simulation Results

**Figure 13:**
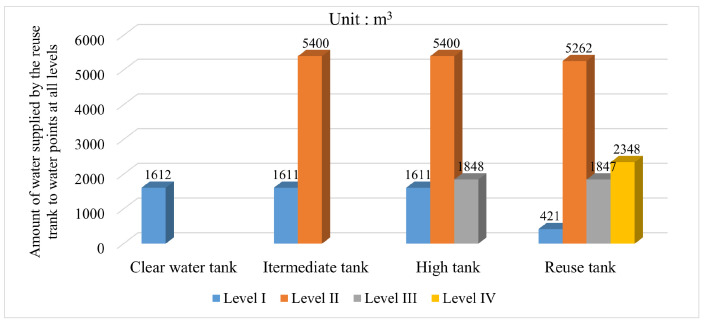
Statistical chart of mine water reuse scheduling in heating season.

**Figure 14:**
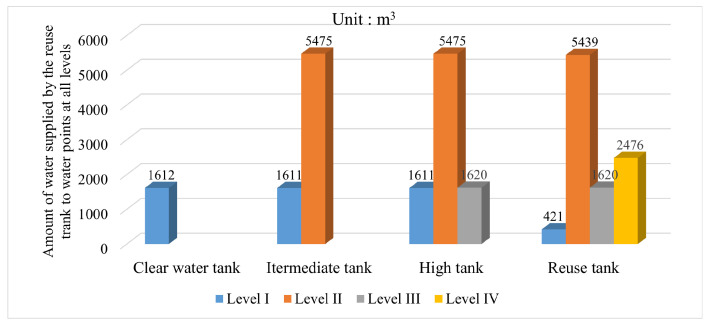
Statistical chart of mine water reuse scheduling in nonheating season.

On the basis of realizing the establishment of the simulation model, this paper conducts an experimental test on the multi-level scheduling of mine water reuse based on the improved whale optimization algorithm. The number of populations was set to 50, and the maximum number of iterations was set to 100. Experiments were carried out under different spatial dimensions of water point scheduling at all levels, and finally, the optimal scheduling scheme of mine water in the heating season and nonheating season was obtained. The results are shown in Figure 13 and Figure 14.

Compared with the traditional reuse method of mine water, which follows the principle of proximity, in the improved multi-level scheduling method, the reuse water of each water point is no longer taken from the reuse tank of its corresponding level alone, but is supplied by multiple reuse tanks at the same time, which realizes the redistribution of water resources between each tank and each water point in the mine water reuse system. Taking a single water point as a reference, it can be found that the water supply from each tank is not equal to each other, which is caused by the combined consideration of mine water reuse cost and reuse time constraints.

In order to verify the superiority of the IWOA in the mine water optimization scheduling problem, this paper also conducted a test using the WOA and compared two test metrics in terms of the objective function value and standard deviation. The results are shown in Table 5.

**Figure 15:**
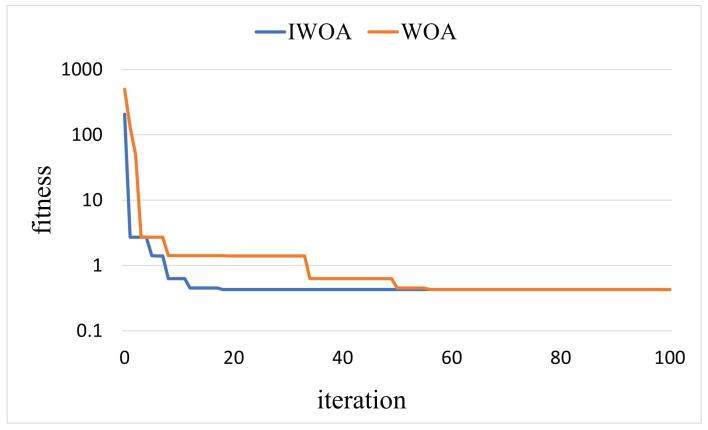
Comparison between the fitness values obtained by the WOA and IWOA during the iterative process.

According to the simulation results, it can be concluded that the objective function value and standard deviation index of the IWOA are better than those of the WOA in multi-dimensional optimal scheduling of two dimensions and above. When the spatial dimension is one, corresponding to the scheduling of level IV water points, due to the limitation of water quality conditions, the reuse water can only be taken from the reuse tank at this time, so there is no scheduling optimization, so its objective function value is 1, and the standard deviation and improvement percentage are both 0. Therefore, the IWOA has a higher convergence accuracy than the WOA. The simulation data for each iteration of the algorithm were recorded, and the simulation figure of the convergence process is shown in Figure 15. It can be observed that the WOA reaches the global optimal solution at about 60 iterations, while the IWOA can reach the global optimal solution at about 20 iterations. Therefore, the IWOA has a faster convergence rate than the WOA.

**Figure 16:**
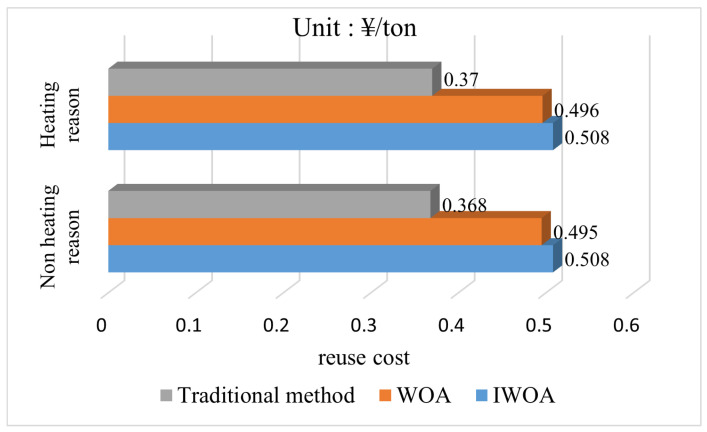
Comparison chart of reuse cost per ton of water.

**Figure 17:**
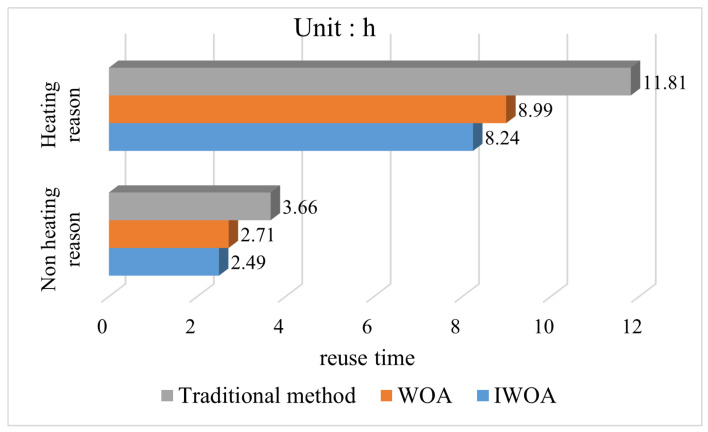
Comparison chart of mine water reuse time.

Based on the obtained optimal mine water scheduling scheme, the mine water reuse time and ton water treatment cost were obtained for the heating and nonheating seasons, where the mine water reuse time is the sum of the water supply time from the reuse tank to the water points at each level in turn. The reuse time and cost of the conventional method and the multi-level scheduling method using the WOA and IWOA seeking optimization were calculated, respectively. The results obtained are shown in Figure 16 and Figure 17 as follows.

According to the content of the comparison chart, in terms of reuse efficiency, compared with the traditional reuse method, the mine water reuse multi-level scheduling method has a significant effect by improving 30.2% in the heating season and 31.9% in the nonheating season. In terms of reuse cost, the tonnage water reuse cost of the multi-level scheduling method of mine water reuse is increased by 27.9% in the heating season and 28.3% in the nonheating season because the improved reuse method has the high-level reuse tank supplying water to the low-level water point. According to the above description in the optimal scheduling model, there is a constraint relationship between reuse time and reuse cost, which will not be optimal at the same time, so an improvement in one will inevitably cause a decrease in the other. In the traditional method, the reuse cost is considered to be more important than the reuse time, so the lowest reuse cost mine water reuse scheme is implemented, but this method cannot achieve efficient mine water reuse deployment. To address this shortcoming, the mine water reuse multi-level scheduling method increases the importance of reuse time to equal that of reuse cost, that is $\omega_{1}=\omega_{2}=0.5$. Therefore, in theory, the degree of the reduction in reuse time should be equal to the degree of increase in reuse cost. The relevant experimental results obtained corroborate this view and demonstrate the correctness of the optimal scheduling model.

For the comparative analysis before and after the algorithm improvement, the reuse efficiency of the IWOA increased by 8.4% in the heating season and 7.8% in the nonheating season compared to the WOA. The cost of tons of water reuse increased by 2.4% in the heating season and 2.6% in the nonheating season compared to the WOA, which shows that the IWOA improves the reuse efficiency in the multi-level scheduling problem of mine water reuse better than the increase of reuse cost. Combined with the conclusion obtained above that the IWOA has higher convergence performance, the superiority of the IWOA can be demonstrated.

## 6. Conclusions

In this paper, a multi-level scheduling method based on the mine water reuse process is proposed to address the inefficiency of mine water reuse. In order to realize efficient mine water reuse deployment, a mine water optimal scheduling model was established and a whale optimization algorithm was used for iterative optimization search. For the possible local convergence defects of the basic whale algorithm, an improvement method based on opposition-based learning, Levy flight, nonlinear convergence factor, and adaptive inertia weighting strategy was proposed. Finally, it was proven through simulation experiments that the IWOA-based mine water reuse optimization scheduling method proposed in this paper can improve the reuse efficiency and equalize the reuse cost with superior convergence performance, which can realize efficient mine water reuse deployment and provide an effective method for solving the mine water reuse optimization problem. In addition, the method has good adaptability to mine water reuse projects in different mining areas.

Considering the influence of other operating conditions, safety performance, and other factors that may exist in the process of mine water reuse, the optimal scheduling model proposed in this paper still has some limitations. In future research, we will seek more optimization objects to reflect a more realistic scenario of mine water reuse. We will also use the theoretical guidance and algorithmic support provided by the scheduling method to realize the development of an automated control system for mine water reuse to complete its application in production practice and further verify its feasibility. In addition, the costs studied in this paper are those incurred due to mine water disposal and do not include the cost impact on mine production due to efficiency improvements. Therefore, the new research topic of the relationship between mine water scheduling and overall coal mine efficiency is also part of our future research program.

## Figures and Tables

**Table 1:** Test functions.

Function	Function Expressions	Range of Values	Optimal Solution
Sphere	$f\left(x\right)=\sum_{i=1}^{d}{x_{i}}^{2}$	[−100,100]	minf=0
Ackley	$f\left(x\right)=-20e^{-0.2\sqrt{\frac{1}{d}\sum_{i=1}^{d}{x_{i}}^{2}}}-e^{\frac{1}{d}\sum_{i=1}^{d}\cos(cx_{i})}+20+e$	[−32,32]	minf=0
Rosenbrock	$f\left(x\right)=\sum_{i=1}^{d-1}\left[100{\left(x_{i+1}-{x_{i}}^{2}\right)}^{2}+{\left(x_{i}-1\right)}^{2}\right]$	[−100,100]	minf=0
Alpine	$f\left(x\right)=\sum_{i=1}^{d}\left|x_{i}\sin\left(x_{i}\right)+0.1x_{i}\right|$	[−10,10]	minf=0
Rastrigin	$f\left(x\right)=\sum_{i=1}^{d}\left[{x_{i}}^{2}-10\cos\left(2\pi x_{i}\right)+10\right]$	[−5.12,5.12]	minf=0
Griewank	$f\left(x\right)=\sum_{i=1}^{d}\frac{{x_{i}}^{2}}{4000}-\prod_{i=1}^{d}\cos(\frac{x_{i}}{\sqrt{i}})+1$	[−600,600]	minf=0

**Table 2:** Comparison of test function results.

Function	Test Indicators	GA	PSO	WOA	IWOA
Sphere	Optimal solution	$3.76\times 10^{3}$	$5.42\times 10^{2}$	$1.43\times 10^{-23}$	$9.40\times 10^{-206}$
	Standard deviation	$3.18\times 10^{3}$	$3.89\times 10^{2}$	$1.05\times 10^{-1}$	0
Ackley	Optimal solution	$1.41\times 10^{1}$	$1.19\times 10^{1}$	$8.25\times 10^{-13}$	$4.44\times 10^{-16}$
	Standard deviation	$1.02\times 10^{0}$	$0.96\times 10^{0}$	$1.94\times 10^{-1}$	$1.48\times 10^{-31}$
Rosenbrock	Optimal solution	$6.16\times 10^{7}$	$3.71\times 10^{6}$	$2.75\times 10^{1}$	$2.85\times 10^{1}$
	Standard deviation	$3.65\times 10^{8}$	$1.08\times 10^{7}$	$1.36\times 10^{0}$	$0.06\times 10^{0}$
Alpine	Optimal solution	$1.58\times 10^{1}$	$0.17\times 10^{0}$	$6.90\times 10^{-15}$	$5.30\times 10^{-127}$
	Standard deviation	$5.12\times 10^{0}$	$1.01\times 10^{1}$	$3.78\times 10^{-1}$	$8.83\times 10^{-87}$
Rastrigin	Optimal solution	$1.73\times 10^{2}$	$3.11\times 10^{1}$	0	0
	Standard deviation	$3.81\times 10^{1}$	$8.42\times 10^{1}$	$1.48\times 10^{0}$	0
Griewank	Optimal solution	$2.08\times 10^{1}$	$1.27\times 10^{0}$	0	0
	Standard deviation	$2.82\times 10^{1}$	$0.13\times 10^{0}$	$0.62\times 10^{0}$	0

**Table 3:** Summary of water resource demand in the mining area.

Types of Water Resources	Location	Water Level	Water Volume in Heating Season (m^3^/d)	Water Volume in Nonheating Season (m^3^/d)
Mine drainage	Underground	\	28,800	28,800
Lowering dust water	Underground	I	3415	3415
Grouting water	Underground	I	1840	1840
Machine dedust water	Ground	II	104	104
Factory supply water	Ground	II	1728	1962.5
Other production water	Ground	II	14,230	14,322.5
Ecological water	Ground	III	2955	2955
Industrial greening water	Ground	III	0	175
Boiler water	Ground	III	740	110
Domestic water	Ground	IV	2130.7	2130.7
Unspecified water	Ground	IV	217.3	345.3

**Table 4:** Process index of mine water reuse treatment.

Tank	Processing Cost (CNY/ton)	Treatment Speed in Heating Season (m^3^/h)	Treatment Speed in Nonheating Season (m^3^/h)
Clear water tank	0.20	$1.36\times 10^{3}$	$4.48\times 10^{3}$
Intermediate tank	0.35	$1.36\times 10^{3}$	$4.48\times 10^{3}$
High tank	0.50	$1.36\times 10^{3}$	$4.48\times 10^{3}$
Reuse tank	0.68	$1.36\times 10^{3}$	$4.48\times 10^{3}$

**Table 5:** Comparison of simulation results before and after algorithm improvement.

Index	Scheduling Type	Spatial Dimension	WOA	IWOA	Increase Percentage
Objective function value	Level I	4	$4.52\times 10^{-1}$	$4.30\times 10^{-1}$	4.87%
	Level II	3	$5.45\times 10^{-1}$	$5.15\times 10^{-1}$	5.50%
	Level III	2	$6.84\times 10^{-1}$	$6.80\times 10^{-1}$	0.58%
	Level IV	1	1	1	0
Standard deviation	Level I	4	$3.01\times 10^{-2}$	$3.56\times 10^{-3}$	88.2%
	Level II	3	$1.98\times 10^{-3}$	$5.59\times 10^{-4}$	71.8%
	Level III	2	$1.59\times 10^{-5}$	$6.44\times 10^{-7}$	95.9%
	Level IV	1	0	0	0

## Data Availability

The data that support the findings of this study are available from the corresponding author upon reasonable request.
